# Supplementary material for: Risk of Mortality from Respiratory Malignant and Non-Malignant Diseases among Talc Miners and Millers: A Systematic Review and Meta-Analysis
Source: Toxics. 2022 Oct 5;10(10):589. doi: 10.3390/toxics10100589 (PMC9612112; doi:10.3390/toxics10100589)
Supplement: Supplementary file 1 [file toxics-10-00589-s001.zip › toxics-1945429-supplementary.pdf]

Risk of Mortality from Respiratory Malignant and Non-Malignant Diseases among Talc Miners and Millers: A Systematic Review and Meta-Analysis

Catalina Ciocan, Alessandro Godono, Sandro Stefanin, Paolo Boffetta, Enrico Pira and Marco Clari

(A)

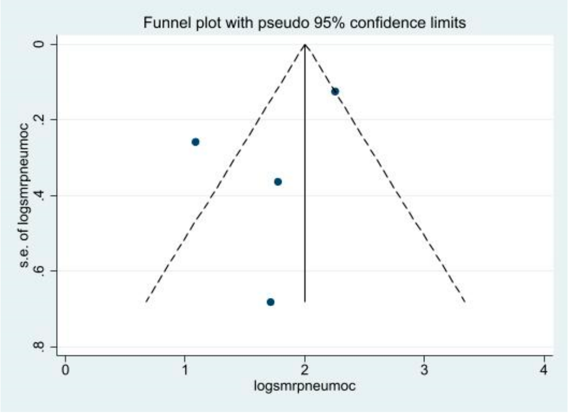

(B)

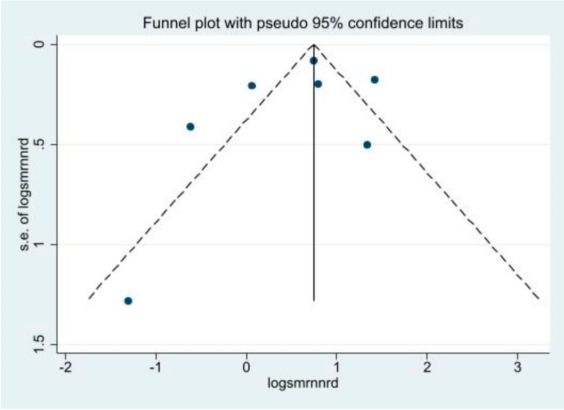

(C)

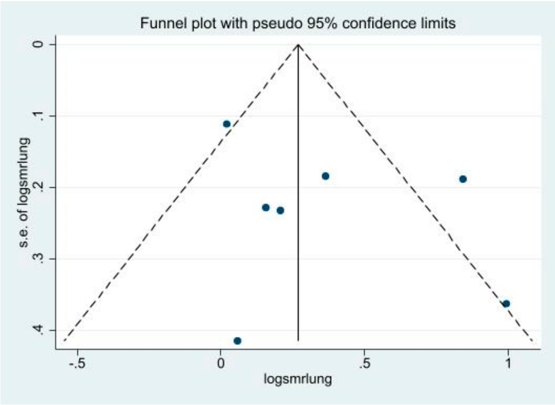

Figure S1. Funnel plot **(A)** pneumoconiosis **(B)** Non malignant respiratory diseases **(C)** Lung cancer.

Table S1. Research String.

Electronic Database	Research String
**Web of Science**	(talc OR talcum) (Topic) AND (miner* OR mining* OR mine*) (Topic) = 2414 risultati, riduced to 63 by filtering for Web of Science Categories: Public Environmental Occupational Health or Medicine Legal
**Scopus**	(TITLE-ABS-KEY ((miner* OR mining* OR mine*)) AND TITLE-ABS-KEY ((talc OR talcum))) = 3873 risultati<br>(TITLE-ABS-KEY ((miner* OR mining* OR mine*)) AND TITLE-ABS-KEY ((talc OR talcum))) AND (LIMIT-TO (SUBJAREA , "MEDI") OR LIMIT-TO

	(SUBJAREA , "PHAR") OR LIMIT-TO (SUBJAREA , "SOCI") OR LIMIT-TO (SUBJAREA , "HEAL") OR LIMIT-TO (SUBJAREA , "NURS")) = 379 risultati<br>(TITLE-ABS-KEY ((miner* OR mining* OR mine*)) AND TITLE-ABS-KEY ((talc OR talcum))) AND (LIMIT-TO (SUBJAREA , "MEDI") OR LIMIT-TO (SUBJAREA , "PHAR") OR LIMIT-TO (SUBJAREA , "SOCI") OR LIMIT-TO (SUBJAREA , "HEAL") OR LIMIT-TO (SUBJAREA , "NURS")) AND (LIMIT-TO (EXACTKEYWORD , "Occupational Exposure") OR LIMIT-TO (EXACTKEYWORD , "Occupational Diseases") OR LIMIT-TO (EXACTKEYWORD , "Occupational Disease") OR LIMIT-TO (EXACTKEYWORD , "Air Pollutants, Occupational")) = 126 risultati<br>(TITLE-ABS-KEY ((miner* OR mining* OR mine*)) AND TITLE-ABS-KEY ((talc OR talcum))) AND (LIMIT-TO (SUBJAREA , "MEDI") OR LIMIT-TO (SUBJAREA , "PHAR") OR LIMIT-TO (SUBJAREA , "SOCI") OR LIMIT-TO (SUBJAREA , "HEAL") OR LIMIT-TO (SUBJAREA , "NURS")) AND (LIMIT-TO (EXACTKEYWORD , "Occupational Exposure") OR LIMIT-TO (EXACTKEYWORD , "Occupational Diseases") OR LIMIT-TO (EXACTKEYWORD , "Occupational Disease") OR LIMIT-TO (EXACTKEYWORD , "Air Pollutants, Occupational")) AND (LIMIT-TO (EXACTKEYWORD , "Mining") OR LIMIT-TO (EXACTKEYWORD , "Miner") OR LIMIT-TO (EXACTKEYWORD , "Coal Mining") OR LIMIT-TO (EXACTKEYWORD , "Miners")) = 69 risultati<br>TITLE-ABS-KEY (("talc miner" OR "talc miners")) = 29 risultati
**Pubmed**	("Miners"[Mesh] OR "Mining"[Mesh]) AND "Talc"[Mesh] = 51 risultati, tutto ciò che ha Pubmed (con i Mesh) sui minatori di talco<br>(miner*[Tiab] OR mining*[Tiab] OR mine*[Tiab]) AND (talc[Tiab] OR talcum[Tiab]) AND (occupational[Tiab] OR worker*[Tiab] OR exposure[Tiab] OR mortality[Tiab] OR morbidity[Tiab] OR cancer*[Tiab] OR tumor*[Tiab] OR tumour*[Tiab] OR neoplasm*[Tiab] OR sarcoma*[Tiab] OR carcinoma*[Tiab] OR asbestos[Tiab] OR asbestosis[Tiab] OR pneumoconiosis[Tiab] OR "silicon dioxide"[Tiab] OR silica[Tiab] OR silicosis[Tiab]) = 172 risultati<br>"Lung Neoplasms"[Mesh] AND "Talc"[Mesh] AND ("Miners"[Mesh] OR "Mining"[Mesh]) = 10 risultati<br>("Asbestos"[Mesh] OR "Asbestos, Amosite"[Mesh] OR "Asbestos, Crocidolite"[Mesh] OR "Asbestos, Amphibole"[Mesh] OR "Asbestos, Serpentine"[Mesh] OR "Asbestosis"[Mesh]) AND "Talc"[Mesh] AND ("Miners"[Mesh] OR "Mining"[Mesh]) = 22 risultati<br>("Talc/poisoning"[Mesh] OR "Talc/toxicity"[Mesh]) AND ("Miners"[Mesh] OR "Mining"[Mesh]) = 11 risultati<br>"Talc/adverse effects"[Mesh] AND ("Miners"[Mesh] OR "Mining"[Mesh]) = 27 risultati<br>("Silicon Dioxide"[Mesh] OR "Silicosis"[Mesh]) AND "Talc"[Mesh] AND ("Miners"[Mesh] OR "Mining"[Mesh]) = 51 risultati<br>("talc miner"[Tiab] OR "talc miners"[Tiab]) = 24 risultati<br>("lung cancer" OR "lung tum*" OR "lung neoplasm*" OR "lung sarcoma" OR "lung carcinoma" OR asbestos OR asbestosis OR pneumoconiosis OR "silicon dioxide" OR silica OR silicosis) AND (talc OR talcum) AND (miner* OR mining* OR mine*) AND (inprocess[sb] OR publisher[sb] OR pubmednotmedline[sb]) = 14 risultati

Table S2. Results reported by the studies in the metanalysis.

**Non-Malignant Respiratory Diseases and Pneumoconiosis**	Fordyce and coll [12] reported 35 deaths for other nonmalignant respiratory diseases (eg, pneumoconiosis and other lung diseases due to external agents) with an SMR=4.13 95% CI 2.87-5.74 although authors sustain that most of the observed deaths for nonmalignant respiratory diseases had prior radiological evidence of pneumoconiosis, with patterns suggesting nonfibrous exposure.<br>Ciocan e coll [11] found significantly increased mortality for pneumoconiosis. This observation was more marked in miners (SMR=12.75; 95% CI 9.80-16.31) than in millers (SMR=2.69; 95% CI 2.24-3.21).<br>Wild and coll [13] attributed the slight excess in the mortality from non-malignant respiratory diseases, in the French cohort, to the significant excess for

	pneumoconiosis (SMR = 5.56; 95% CI 1.12 – 16.2). However, mortality from non-malignant respiratory diseases was lower than expected in the Austrian cohort.<br>Dement et coll [26] found an excess mortality from non-malignant respiratory diseases (SMR = 3.80, calculated 95% CI = 1.249-8.976, p<0.05) but Authors did not evaluate mortality for pneumoconiosis.<br>The total number of deaths from non-malignant respiratory diseases, excluding pneumonia and influenza, in the Norwegian cohort [15] was lower than expected (SMR = 0.54; 95% CI 0.22 – 1.11). There were 2 cases of pneumoconiosis (silicosis) as contributory causes of death, one miner and one miller.<br>Honda and coll [27] found that the overall increase in NMRD deaths was not limited to a particular form of respiratory disease but was greatest for other NMRD (COPD and fibrosis) (SMR = 2.91, CI = 1.73–4.75 with 1 case of asbestosis, 5 cases of pneumoconiosis and 1 case of chronic pulmonary fibrosis.
**Lung Cancer**	In a review published in 2006 [7], Wild P analyzed the risk for lung cancer in workers occupationally exposed to talc (talc mine, rubber industry, ceramic industry, glass fiber production) and found no excess risk for lung cancer in talc miners and millers. There was an excess risk in the ceramic industry and rubber industry.<br>Another review and meta-analysis [16] included both talc-producing and talc user industries. After subgrouping for industry type, the meta-SMR of the talc-producing industry was 1.47 (95%CI: 1.02-2.10; p=0.0372). The meta-analysis included the Chinese cohort [24]. Unfortunately, it was not possible for us to have access to the full paper (although in Chinese) nor the abstract therefore data from this study is not included.<br>Katsnelson's study [33], not included in the meta-analysis because of the poor quality score (score 6), found excess mortality for lung cancer in the Russian talc cohort, in men as well as in women (SMR men 4.5, no CI 95% reported, SMR women 9.3, no CI 95% reported). This result is very difficult to explain as Authors stated that men were employed in the talc mine for a much longer period of time than women and that the prevalence of smokers in men was above 66-75% while in women was 0-1.5%, two conditions that would determine a higher mortality rate for lung cancer in men than in women. Also, in this study, results showed that mortality for lung cancer in workers exposed to talc was higher than workers exposed to asbestos, which is not consistent with other findings.<br>Fordyce et coll [12] found a borderline, non-significant excess of lung cancer based on a two-sided statistical test of significance. A one-sided test was significant at the 0.05 level; however, there was no indication of a trend in risk with increasing duration of employment, suggesting that the borderline increase in risk could be attributed to factors other than exposure in the mines and mills.<br>Ciocan et coll [11] stated that throughout the follow-up of the Italian cohort there was no excess mortality from lung cancer in either miners or millers. The data confirmed that exposure to talc with no detectable level of asbestos is not associated with the risk of this disease.<br>Mortality from lung cancer in the French and Austrian cohorts [13] showed a slight non-significant excess in both cohorts. Same observation in the Norwegian cohort [15].<br>Available data on tobacco smoking in the observed cohorts show that the prevalence of current smokers was similar to that of men in the reference countries. Therefore, the lack of excess cannot be explained by the lower prevalence of smokers.<br>Dement et coll [26] found an excess mortality from lung cancer (SMR = 2.73, calculated 95% CI = 1.247-5.177, p<0.01). All subjects were exposed to both

	asbestiform tremolite and anthophyllite but little free silica. Authors stated that several possible confounding factors must be taken into consideration before attributing this mortality pattern to talc exposure (smoking, prior employments, etc) but they concluded that exposures to asbestiform tremolite and anthophyllite stand out as the prime suspected etiologic factors associated with the excess mortality from bronchogenic cancer.<br>The study of Honda et al [27] showed that increased mortality from lung cancer was limited to men hired before 1955. This subgroup had 28 lung cancer deaths compared with 9.8 expected (SMR = 286, CI = 190–414), whereas men hired in or after 1955 had three deaths compared with 3.6 expected.The overall excess of lung cancer was concentrated among men ever employed in the mines.<br>The lung cancer excess in the overall study group was moderately strong and was concentrated in the follow-up period 20 or more years since hire, results that suggest that some aspect of employment at the plant may have been associated with lung cancer. of employee controls had been smokers. It is unlikely that the high smoking prevalence (73%) could explain the entire moderately strong lung cancer excess, particularly the nearly 4-fold increase among miners.
**Mesothelioma**	Regarding malignant mesothelioma, only one cohort (Fordyce 2019) reported data (SMR=1.004, 95%CI =0.12-3.626).<br>There was no case of mesothelioma reported in the cohorts from Italy [11], France, and Austria [13]. Pira et al.[10] reported two observed deaths from peritoneal cancer, other than peritoneal mesothelioma, and no case of pleural mesothelioma. The long follow-up provides evidence against the hypothesis that the lack of deaths from mesothelioma can be attributed to insufficiently long latency.<br>Fordyce and coll.[12] reported one case of mesothelioma after the reanalysis of the death certificates by the trained nosologist, but no SMR was reported. There was another death for mesothelioma, in a previous follow-up of the cohort, reported by Lamm and Starr [26], before 1975.<br>Dement et coll [26] reported one death due to mesothelioma in the study population. The individual worked for 11 years in construction work, previously.<br>Honda et al [27] found two deaths from mesothelioma. Authors stated that because of the short latency for the first case and the low talc exposure of the second case, it is unlikely that either of the two mesotheliomas was due to talc ore dust. The two cases could be more likely explained by asbestos exposure during past employment.
**Exposure levels evaluation.**	Exposures in the talc operation, before the adoption of technical preventive measures in 1950, in Piedmont, Italy, were reported to be approximately 800 mppcf in the mines and 25 mppcf in the mills. Exposures after 1965, when improved ventilation techniques and wet drilling procedures were introduced, were reduced to less than 10 mppcf. Lately (after 2003), the mean exposure to respirable dust was 0,01 mg/m3 (range, 0.5–2.5 mg/m3), while the mean exposure to talc alone was 1.0 mg/m3 (range, 0.03–0.02 mg/m3). Medium quartz level was 10% before 1974, 2% in 1978 while, in recent years (after 2003), it was below 1% (Pira 2017). There is strong evidence that talc from the Piedmont mine is not contaminated by asbestos. [8, 9, 10, 11, 31]<br>Wild et coll (13, 32) reported exposure in a French talc producing factory and in three Austrian mines and their respective mills in the Styrian Alps.<br>For France, the extracted ore consisted of a mixture of talc, chlorite, some dolomite (<3%), occasionally quartz (<3%), and traces of calcite, apatite, pyrite, and mica.

	Amphiboles were not detected. Between 1986 and 1991 the mean levels of exposure to respirable dust ranged from 0.5 mg/m3 to 15 mg/m3.<br>For Austria, at site B, the ore contained between 0.5 and 4% quartz. At site C, the amount of quartz in the end-product was below 1%. However, materials from certain parts of this mine that were rich in dolomite could have contained 2–3% quartz.<br>The concentration for total suspended particles was 6.14 mg/m3. Further analyses of the samples with PM <10 μm and <2 μm by scanning electron microscopy showed that the fiber concentration was 0.25 fibers/cm3 and 0.12 fibers/cm3, respectively. Analyses by polarized light microscopy indicated the presence of asbestiform tremolite, chrysotile, and anthophyllite in these samples.<br>Dement et coll [26] reported concentrations of respirable dust in mass samples from a Vermont talc mine and mill. Free silica exposure was very low: 0.040 mg/m3 while respirable dust exposures ranged from 0.25 to 2.96 mg/m3. The talc from this mine contains fibrous tremolite and anthophyllite.<br>The estimated exposures of an expanded cohort in Vermont [27, 34] were validated by comparing them with available measured historical exposure concentration. The estimated average respirable dust concentration ranged from 0.01–2.67 mg/m3.<br>Wergeland e coll [15] reported that total dust levels from personal samples measured from 1980 to 1982 varied, by work operation and worksite, with a basal level ranging from 0.94 to 97.35 mg/m3 and with peak exposures during drilling operations of 318.9 mg/m3. Repeated exposure measurements after 1981 showed only trace amounts of tremolite and anthophyllite and trace amounts of quartz. For miners, there were reported peak levels of quartz of 3-6% during drilling operations.
